# No Surprises Act arbitrators vary in provider win rates and payments

**DOI:** 10.1093/haschl/qxag265

**Published:** 2026-09-28

**Authors:** Philip Valtadoros

**Affiliations:** Department of Economics, The University of Texas at Austin, Austin, TX 78712, United States

**Keywords:** No Surprises Act, arbitration, out-of-network billing, surprise billing

## Abstract

**Introduction:**

The No Surprises Act (NSA) banned balance billing and established an arbitration process, known as independent dispute resolution (IDR), for insurers and out-of-network providers to resolve payment disputes over out-of-network services. Publicly available IDR data only recently included arbitrator identity and still lack information about arbitrator selection.

**Methods:**

Using novel data documenting arbitrator identities and the manner in which they were selected for cases filed in 2023 obtained in a Freedom of Information Act request, I document substantial variation across arbitrators in provider win rate, payment, wait time, case volumes, and selection methods.

**Results:**

The arbitrators with the highest and lowest provider win rates differed by 48 percentage points in the fraction of cases in which they choose the provider's offer. Arbitrators differ in how they are selected to decide cases; disputing parties differ in the selection methods they use to select arbitrators.

**Conclusion:**

The way arbitrators are selected is an important, yet unobserved dimension of arbitration under the NSA.

## Introduction

The No Surprises Act, enacted in 2020 and implemented in 2022, banned surprise billing^1,2^ and set up an arbitration process for providers and insurers to resolve disputes over eligible out-of-network services. Under this dispute resolution process, providers and insurers jointly choose or are assigned 1 of 13 arbitrators, both submit offers, and the arbitrator chooses one or the other. Prior work has documented high provider win rates and payments in this dispute resolution process^3-5^ but has paid limited attention to the role of the arbitrators due to a lack of information. Using newly obtained data in a Freedom of Information Act (FOIA) request, I document variation across arbitrators in case outcomes, case volumes, and methods of selection.

Publicly available case-level data from the Centers for Medicare and Medicaid Services (CMS) contain no information about how arbitrators are chosen. The CMS made arbitrator identities available in the most recent release of public use files for cases decided in the second half of 2025,^6^ but prior files do not. For cases decided before July 1, 2025, it is possible to partially infer arbitrator identity using the fee charged in some cases,^7,8^ but this provides incomplete identification. In each case, the provider and insurer each have an opportunity to propose an arbitrator and if both reject the other's proposal then one is randomly selected (simple random assignment).^9^ Using data on arbitrator identity and the method of selection for cases initiated in 2023, I document wide variation in case outcomes, volumes, and selection methods across arbitrators. The arbitrators with the highest (EdiPhy Advisors, LLC [EdiPhy]) and lowest (Keystone Peer Review Organization [Keystone]) provider win rates differ by 48 percentage points in the fraction of cases in which they choose the provider's offer. The arbitrators with the highest (C2C Innovative Solutions [C2C]) and lowest (National Medical Reviews, Inc [NMRI]) mean payments differ by 360% of the insurer's median in-network rate. Arbitrators also vary widely in how many cases they were selected to decide. The arbitrator with most cases (Federal Hearings and Appeals Services, Inc [FHAS]) was assigned 101 149 cases, while the arbitrator with the fewest (Keystone) was assigned only 11 312 cases. Arbitrators with high provider win rates also decide a larger share of their assigned cases, have larger case volumes, and larger shares of cases selected by the provider alone, although these patterns are not uniform. I also document that providers and insurers engage with the selection process with varying intensities.

Failure to account for arbitrator agency gives a partial picture of the dispute resolution process. Differences in case outcomes could reflect differences in the case characteristics of each arbitrator's set of cases or in decisions. Previous and ongoing research acknowledges but is unable to appraise the importance of arbitrators and the selection process because information is scarce. Despite their characterization as impartial and identical third parties in the canonical arbitration literature,^10-14^ arbitrators—which are firms or nonprofits—have agency.

## Data and methods

I used 2 data sources from the CMS to investigate arbitrator differences in case outcomes and volumes. The first is the public use data published by CMS that contain case outcome, disputing party identities, and arbitrator fees for all cases decided between January 1, 2023, and December 31, 2025.^15^ These data are given at the line-item level (a dispute can contain more than 1 line item if services are batched together). These data contains 3 480 851 disputes and 8 329 632 line items.

The second is an unpublished dataset obtained using a FOIA request that contains arbitrator identity, dispute identifiers, initiating party identity (provider or insurer), and selection method information for all disputes initiated in 2023. (Appendix Table A1 shows arbitrator revenue, organizational structure, and parent organizations.) I verified FOIA de-anonymized arbitrator identifiers using the arbitrator identities in the public use files released for the second half of 2025. The set of cases initiated in 2023 and decided in the second half of 2025 is small, but all arbitrator identifiers match. (Appendix Table A2 provides a cross-tabulation of the most recent public use file identifiers and FOIA identifiers for the small subset of cases initiated in 2023 and decided in the second half of 2025.) These data are merged to the public use case outcome data using dispute identifiers. A total of 517 734 decided cases (including 893 725 unique line items) match to the 677 137 cases initiated. (Appendix A presents technical details regarding this merge.)

Case volumes are calculated using the counts of initiated disputes; case outcomes are calculated using the results from decided disputes. The main outcomes of interest are case volumes for each method of selection (dispute level), provider win rate (line-item level), payment in terms of the insurer's median in-network rate or Qualifying Payment Amount (QPA) (line-item level), and wait time in days (dispute level). Payment in terms of the QPA is winsorized at the 99th percentile of line items to mitigate the influence of outliers. Table 1 presents the tabulation of methods of selecting arbitrators for cases initiated in 2023. Appendix Figure A1 presents the shares of each selection method by quarter of initiation. The fraction of cases in which an arbitrator is randomly assigned in quarter 4 is more than double the fraction in quarter 1. Figure 1 presents a flow chart describing the selection process. For a detailed explanation of the selection process, see Appendix B.

**Figure 1.**
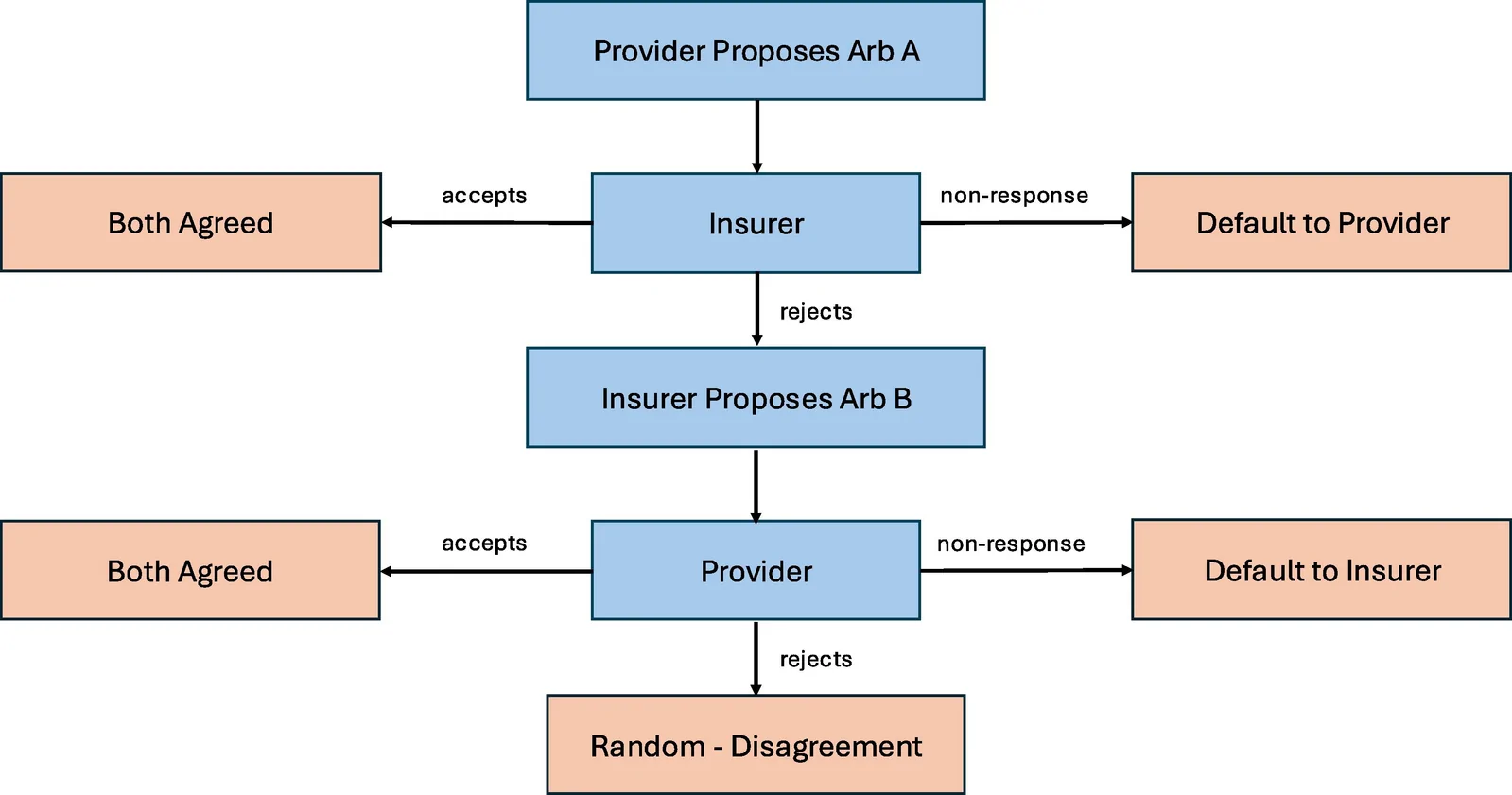
Arbitrator selection flowchart. The flowchart shows the sequential proposal-rejection arbitrator selection process. The provider initiates over 99% of cases and proposes an arbitrator first. The insurer can accept, reject, or not respond to the proposal: if she accepts, the two have both agreed on an arbitrator; if she does not respond, the arbitrator is chosen by a default to the provider’s proposal; if she rejects, then she has a chance to propose an arbitrator. The provider then has the same three options. If the provider rejects the insurer’s proposal, an arbitrator is randomly assigned. Source: The arbitrator selection process is taken from CMS’ guidance for certified IDR entities. Default to none is omitted from the figure. This occurs when neither party proposes an arbitrator. Abbreviations: Arb, arbitrator; CMS, Centers for Medicare and Medicaid Services; IDR, independent dispute resolution.

**Table 1.** Tabulation of methods used to select arbitrators in 2023.

Arbitrator selection method	Frequency	Percent
Both agreed	246 144	36
Default to insurer	42 416	6
Default to none	1	0
Default to provider	176 123	26
Random—COI	6465	1
Random—disagreed	205 988	30
Total	677 137	100

Abbreviations: CMS, Centers for Medicare and Medicaid Services; COI, conflict of interest; FOIA, Freedom of Information Act.

Source: Author's analysis of CMS arbitrator identity and assignment data obtained using FOIA. Selection methods and their meanings: Both agreed indicates that 1 party proposed and the other accepted an arbitrator; Default to insurer indicates that the insurer proposed and the provider did not respond, resulting in default selection of an arbitrator; Default to none indicates that neither party proposed an arbitrator, resulting in random selection of an arbitrator; Default to provider indicates that the provider proposed and the insurer did not respond, resulting in default selection of an arbitrator; Random—COI indicates that the arbitrator initially selected attested to a conflict of interest with either provider or insurer, recused itself, and an arbitrator was randomly selected from the remaining pool; Random—disagreed indicates that both parties rejected the other's proposal and an arbitrator was randomly assigned (simple random assignment).

These arbitration data have several limitations. Differences across arbitrators in case outcomes are not causal estimates. Disputing parties can choose an arbitrator for each dispute and each arbitrator's case composition may differ. (Appendix C provides a full discussion of case composition, selection, and offers.) A large share of initiated cases in the FOIA data do not match to any case decisions in the public use file. Appendix Table A3 shows that the number of cases initiated in 2023 that receive decisions in the last quarter of 2025 is 809. It is therefore unlikely that many of the cases without decisions remain in queue. I did not observe characteristics of these cases, including reasons for non-decision. All 13 arbitrators were certified for the duration of 2023 and there is limited evidence that arbitrators were available to accept cases for most of the year, but capacity and availability are ultimately unobserved. (Appendix D provides a full discussion of tenure, availability, and capacity.)

## Results

Table 2 shows substantial arbitrator variation in provider win rates, payments, wait times, case volumes, and methods of selection. Provider win rates range from 45% (Keystone) to 93% (EdiPhy Advisors), where the overall mean is 81%. Payments range from 340% of the insurer's QPA for NMRI to 700% for C2C. Wait times range from 47 days for FHAS to 300 days for MCMC Services (MCMC).

**Table 2.** Arbitrator-level case outcomes and case volumes, in total and by selection method in 2023.

Arbitrator	Provider win rate, %	Payment as % of QPA	Wait time, days	Total cases, *n*	Decision rate, %	Random, %	Jointly agreed, %	Default to provider, %	Default to insurer, %
Keystone	45	610	245	11 312	56	0	32	6	62
MET	82	534	157	78 456	75	19	41	38	1
PRI	60	361	120	38 806	71	57	33	8	1
PPR	74	461	97	25 160	74	87	6	3	1
NMRI	70	340	155	54 287	68	35	38	22	4
NMRC	74	411	281	55 906	78	39	40	19	1
MFS	70	503	207	41 216	74	47	17	8	27
EdiPhy	93	508	184	43 207	87	38	34	24	2
MCMC	91	478	300	66 282	82	26	46	24	3
FHAS	87	635	47	101 149	85	9	44	46	0
C2C	81	700	102	77 173	71	19	36	24	21
MPRO	88	386	149	34 859	85	52	32	11	3
IPRO	89	529	223	49 324	68	20	36	42	1

Abbreviations: CMS, Centers for Medicare and Medicaid Services; COI, conflict of interest; FOIA, Freedom of Information Act.

Source: Author's analysis of CMS independent dispute resolution public use files and CMS arbitrator identity and assignment data obtained using FOIA. Arbitrator abbreviations correspond to entity names as follows: Keystone, Keystone Peer Review Organization, Inc; MET, Medical Evaluators of Texas; PRI, Provider Resources, Inc; PPR, ProPeer Resources, LLC; NMRI, National Medical Reviews, Inc; NMRC, Network Medical Review Company; MFS, Maximus Federal Services, Inc; EdiPhy, EdiPhy Advisors, LLC; MCMC, MCMC Services, LLC; FHAS, Federal Hearings and Appeals Services, Inc; C2C, C2C Innovative Solutions Inc; MPRO: iMPROve Health, formerly Michigan Peer Review Organization; IPRO, Island Peer Review Organization. The first 3 columns are case outcomes. Provider win rate is the percentage of line items in which the provider won. Payment as % of QPA is the payment amount represented in terms of the insurer's Qualifying Payment Amount (the median in-network rate for the same service, same geographic region) and is winsorized at the 99th percentile of line items. Both win rate and payment are line-item-level outcomes. Wait time is the number of days from initiation to decision and is a dispute level outcome. Decision rate is the percentage of initiated cases that were and decided by the selected arbitrator. Random, jointly agreed, default to provider, and default to insurer are the percentage of an arbitrator's cases selected via each method. Selection methods “Default to none” and “Random—COI” are omitted from this table because very few arbitrators are selected with either of these methods (<1% of cases use these methods).

Arbitrators also varied in case volumes and in how they were selected to decide cases. Individual arbitrators were selected to decide as few as 11 312 (Keystone) and as many as 101 149 (FHAS) cases. Arbitrators vary in the fraction of cases they decide. Keystone has the lowest decision rate at 56% and EdiPhy has the highest at 87%.

Arbitrators vary in how they are selected to decide cases, including in how many of their cases were randomly assigned. FHAS is chosen most often by joint agreement between the provider and insurer (44 657 cases), while ProPeer Resources is chosen the least (1480). FHAS, which administers the highest payment and shortest wait times, is also the most frequently chosen arbitrator by default to the provider (46 419). The provider's proposal is accepted by default if the insurer does not reject or accept its arbitrator proposal. C2C Innovative Solutions is most frequently chosen by default to the insurer (15 814). Interestingly, C2C also administers the highest payment and relatively short wait times. Finally, arbitrators differ in their randomly assigned case counts, from 22 (Keystone) to 22 175 (Provider Resources).

These differences in case outcomes might result from disputing party offering behavior, case compositions, disputing party selection, or other factors. Appendix Table A4 presents the provider and insurer offers as percentages of the QPA and payment in dollars. Appendix Tables A5–A9 present case outcomes and volumes split by the following broad service categories: radiology, emergency, anesthesia, air ambulance, and other services. Appendix Table A10 presents definitions of these categories. Appendix Table A11 presents case outcomes and decision rates split by random (random—disagreed or random—conflict of interest) and nonrandom assignment. In all splits of the data, wide variation in case outcomes persists: the gradient in provider win rate and payment that is present in Table 2 remains.

Table 3 shows the volume of cases initiated and methods of selection for the 10 highest volume providers and all others combined and the 9 highest volume insurers and all others combined. Providers differ in how active a role they play in selecting arbitrators. TeamHealth, the highest volume provider group, sees over half of its cases randomly assigned to an arbitrator, indicating that both TeamHealth and its insurer opponents frequently reject each other's arbitrator proposals. TeamHealth's 52% is among the highest rate of randomly assigned cases and is higher than the 23% of other providers, who are typically much smaller entities. Additionally, TeamHealth has fewer than 1% of its cases chosen by the insurer by default. TeamHealth engages more frequently in the proposal process, whereas other providers do not respond to an opponent's proposal in 10% of cases.

**Table 3.** Selection methods used by large providers and insurers in 2023.

	Total cases, *n*	Jointly agreed, %	Default: provider, %	Default: insurer, %	Random COI, %	Random disagreed, %
Provider						
TeamHealth	147 950	28	19	1	0	52
AGS Health	54 292	37	29	5	0	28
SCP Health	50 520	32	29	13	0	25
Radiology Partners	48 523	31	40	1	1	27
Envision	38 459	61	11	1	0	28
Specialty Care	15 897	35	39	4	0	21
HaloMD	11 722	53	28	9	0	11
Total Care	9573	32	41	3	0	24
Sonoran Radiology	8335	18	27	1	0	54
R1RCM	4803	51	28	22	0	0
Other	287 063	38	27	10	2	23
Insurer						
UnitedHealthcare	172 652	42	4	3	1	51
Anthem	93 735	22	34	9	1	35
Multiplan	65 845	9	38	10	1	42
Aetna	64 688	77	22	1	0	0
BCBS TX	22 490	40	57	1	0	1
BCBS TN	20 456	2	90	6	0	2
Cigna	11 987	31	19	11	2	38
BCBS IL	11 870	51	47	1	0	0
BCBS NC	4162	70	10	6	0	14
Other	209 252	36	28	9	1	25

Abbreviations: BCBS, Blue Cross Blue Shield; CMS, Centers for Medicare and Medicaid Services; COI, conflict of interest; FOIA, Freedom of Information Act; IL, Illinois; NC, North Carolina; TN, Tennessee; TX, Texas.

Source: Author's analysis of CMS independent dispute resolution public use files and CMS arbitrator identity and assignment data obtained using FOIA. The table includes the 10 providers groups and 9 insurers with the most disputes (initiated in 2023) decided. Initiated, undecided cases do not have provider or insurer identity and are included under Other categories. Selection methods “Default to none” and “Random—COI” are omitted from this table because no more than 2% of any high-frequency provider or insurer's cases use these methods.

Insurers vary in their methods of selecting arbitrators. United Healthcare, the highest-volume insurer, has the highest rate of randomly assigned cases (51%) and the lowest rate of provider chosen by default cases (4%). Many of the large insurers allow a large share of provider opponents to choose by default. Blue Cross Blue Shield (BCBN) of Tennessee does not respond to provider proposals in 90% of cases, while the remaining insurers do not respond in 4%–57% of cases. Differences in provider and insurer behavior with respect to each other's arbitrator proposals indicate that provider groups less often accept by default their opponent's arbitrator proposal.

The selection methods used by each provider and insurer are dependent on their opponents’ acceptance and rejection decisions. An arbitrator will be randomly assigned only if both parties reject the other's proposal. Appendix Table A12 presents the methods of selection for the 19 highest volume provider–insurer pairs and for all other pairs. Provider–insurer pairs that include disputing parties with high random assignment rates (TeamHealth, UnitedHealthcare) also have frequent random assignment. TeamHealth and UnitedHealthcare have low rates of default selections (2% by provider, 1% by insurer) and the second highest rate of random assignment among high-volume pairs. Less-active parties such as BCBS of Tennessee, BCBS of Texas, or Aetna have high rates of joint agreement or default selections by providers. TeamHealth and BCBS of Tennessee, TeamHealth and Aetna, and Total Care and BCBS of Texas select an arbitrator proposed by the provider in over 99% of their cases.

## Discussion

Arbitrators vary widely in case outcomes, case volumes, and the method by which they are selected. These patterns are important for understanding patterns in overall case outcomes and volumes. Only in the last release of public use files was arbitrator identity made available. Selection methods remain unobservable in these data. Some arbitrators who administer relatively high provider win rates decide a higher share of cases they are assigned, have higher case volumes, and in larger (smaller) shares of cases they are chosen by the provider (insurer) by default, although not uniformly.

The FHAS has one of the highest provider win rates, the second highest average payment, and the shortest average wait time. It also has the largest case volume; 45.9% of its cases are chosen by the provider by default, and only 0.3% are chosen by the insurer by default. However, other arbitrators with higher provider win rates (iMPROve Health, formerly Michigan Peer Review Organization; MCMC Services, LLC) or higher average payment (C2C) decide fewer cases and fewer (more) cases where they are chosen by provider (insurer) default. Win rate, payment, and wait time may not be the only factors that disputing parties consider when selecting an arbitrator. At the time of choice, either or both parties may not have known which arbitrators would favor them. In a case where the arbitrator was chosen by default by provider, the insurer may have tacitly consented or may have failed to actively select an arbitrator. We cannot distinguish between choice, incomplete information, and inaction.

Arbitrators who decide large shares of their cases also have high provider win rates. Keystone has both the lowest provider win rate (45%) and the lowest decision rate (56%). EdiPhy has both the highest provider win rate (93%) and the highest decision rate (87%). This pattern holds broadly but not uniformly; Island Peer Review Organization has a high provider win rate (89%) but a below-median decision rate (68%). Even among random cases (Appendix Table A11), arbitrators with higher win rates have higher decision rates. If arbitrators systematically differ in their filtering of ineligible cases, their case outcomes could be affected. Unfortunately, case characteristics including reason for dismissal or non-decision are not observed.

Arbitrators have different randomly assigned case volumes. According to regulatory documents, this assignment is simple random assignment, but this does not align with the observed differences in random case volumes. I did not observe the availability and capacity of each arbitrator, which might be able to explain this asymmetry.

Providers and insurers also differ in how they select their arbitrators. In general, insurers accept their opponent's proposal by default more often than providers do. These patterns may highlight differences between providers and insurers in how actively they engage with the selection process.

## Supplementary Material

qxag265_Supplementary_Data

## Data Availability

The Independent Dispute Resolution Reports are publicly available on CMS' website at https://www.cms.gov/initiatives/no-surprise-billing/overview/policies-resources/independent-dispute-resolution-reports. Data obtained via FOIA cannot be made public, but can be accessed via CMS' FOIA portal: https://www.foia.gov/request/agency-component/e07b59b9-db3b-4611-898d-23b42f7ead8d/.
