# Supplementary material for: No Surprises Act arbitrators vary in provider win rates and payments
Source: Health Aff Sch. 2026 Sep 28;4(10):qxag265. doi: 10.1093/haschl/qxag265 (PMC13711659; doi:10.1093/haschl/qxag265)
Supplement: Supplementary file 1 [file qxag265_supplementary_data.zip › qxag265_Supplementary_Data.pdf]

Appendix Material for:

# No Surprises Act Arbitrators Vary in Provider Win Rates and Payments

Philip Valtadoros

**This file includes:**

- Appendix Text, Sections A to D
- Appendix Tables A1 to A12, Appendix Figure A1
- Appendix References

**Other supporting materials for this manuscript include the following:**

- The Independent Dispute Resolution Reports are publicly available on CMS' website at cms.gov.
- Data obtained via FOIA cannot be made public, but can be accessed via CMS' FOIA portal: foia.gov.
- National Plan and Provider Enumeration System (NPPES) file publicly available on CMS' website at cms.gov.
- Zip to CBSA crosswalk publicly available on HUD's website at huduser.gov.
- CBSA file publicly available on the EPA's website at epa.gov.

# Contents

**Appendix Text** **3**

A. Data Construction and Merge Procedure . . . 3

B. Arbitrator Selection Process and Selection Methods . . . 4

C. Additional Information on Case Outcomes, Case Volumes, and Methods of Selection . . . 5

D. Arbitrator Tenure, Availability, and Capacity . . . 6

**Appendix Tables** **7**

Appendix Table A1: Arbitrator Revenues and Corporate Structures . . . 8

Appendix Table A2: FOIA Arbitrator Identifiers Matched to CMS PUF Arbitrator Identifiers . . . 9

Appendix Table A3: Tabulation of Quarter and Year of Decision for Cases Initiated in 2023 . . . 10

Appendix Table A4: Payments, Offers, and Fees by Arbitrator . . . 11

Appendix Table A5: Case Outcomes and Volumes by Arbitrator for Radiology Services . . . 12

Appendix Table A6: Case Outcomes and Volumes by Arbitrator for Emergency Services . . . 13

Appendix Table A7: Case Outcomes and Volumes by Arbitrator for Anesthesia Services . . . 14

Appendix Table A8: Case Outcomes and Volumes by Arbitrator for Air Ambulance Services . . . 15

Appendix Table A9: Case Outcomes and Volumes by Arbitrator for Other Services . . . 16

Appendix Table A10: Broad Service Category Definitions . . . 17

Appendix Table A11: Case Outcomes by Random/Non-Random Arbitrator Selection . . . 18

Appendix Table A12: Selection Methods Used by Large Provider-Insurer Pairs in 2023 . . . 19

**Appendix Figures** **20**

Appendix Figure A1: Selection Methods by Quarter of Filing . . . 20

**Appendix References** **21**

# Appendix Text

## A. Data Construction and Merge Procedure

I obtained arbitration identity and selection method information from Centers for Medicare  Medicaid Services (CMS) using a Freedom of Information Act Request submitted on January 16, 2025. I received a data extract on May 21, 2026 including anonymized arbitrator identity, initiating party type (ie provider or insurer), non-initiating party type, and selection methods for 677,137 cases identified using their unique dispute numbers. No documentation for this dataset was provided. No information regarding dates of filing was provided either.

I match anonymized arbitrator identifiers to entity names using the fees charged in disputes that merge with the data obtained via FOIA. All 13 arbitrators have a unique pair of single and batched fees, and this allows me to name all 13. For example, if I see anonymous arbitrator 9 charge $395 in single disputes and $660 in batched disputes, I know with certainty that this is MCMC Services, even though Federal Hearings and Appeals Services charges the same fee in single disputes (but a different fee in batched filings). See Appendix Table A1 for arbitrator revenue, organizational structure, and parent organizations. See Appendix Table A2 for a cross tabulation verifying these FOIA identifiers against the arbitrator identities disclosed in the public use files released for the second half of 2025. The set of cases initiated in 2023 and decided in the second half of 2025 is small, but 2,187 of 2,189 (99.9%) identifiers match.

Initiating and non-initiating party types took one of the following values: Federal Employees Health Benefits Carrier, group health plan, individual health insurance issuer, health care facility, health care provider, or provider of air ambulance services. I classify the first 3 as insurers and the latter 3 as providers.

The selection method by which the arbitrator is chosen is given as one of the following values: Both Agreed, Default to Initiating Party, Default to Non-Initiating Party, Default to None, Random – COI, or Random – Disagreed. Using the types of initiating and non-initiating parties, I translate "Default to Initiating Party" into "Default to Provider" if the initiating party is a provider or into "Default to Insurer" if the initiating party is an insurer. Similarly, I translate "Default to Non-Initiating Party" into "Default to Provider" if the non-initiating party is a provider or into "Default to Insurer" if the non-initiating party is an insurer.

These cases have dispute numbers ranging from 200,502 to 880,169. The difference between 880,169 and 200,502 is 679,667. There are 677,137 cases in the FOIA records. According to Congressional Reports 200,112 cases were initiated in 2022 and 679,156 cases were initiated in 2023 [S1]. The reported number of initiated cases in 2022 is very close to the beginning dispute number in the data and the reported number of initiated cases in 2023 is similar to the number of cases provided to me by CMS. If cases are numbered in the order in which they are received, then it is plausible that the cases numbered from 200,502 to 880,169 are the cases initiated in 2023. Further confirmation that these cases are filed in 2023 is provided by the fees arbitrators charge in these cases. 92% of these cases have an arbitrator fee that was charged in 2023 [S2]. Due to the incomplete merge to 2023 case decisions, the sequence of dispute numbers, and the fact that the beginning and ending dispute numbers nearly match the total year end dispute counts, I conclude that the data is for 2023 initiations.

Of the 677,137 unique disputes assigned to arbitrators in 2023, 517,734 match to dispute outcomes in the public use file and 159,403 do not. Two potential reasons for this incomplete merge are that some cases remain under review long after being assigned, are withdrawn by the initiating party, or are dismissed by the arbitrator after being selected. Appendix Table A3 presents the tabulation of quarter of decision for cases initiated in 2023. The number of cases initiated in 2023 that were decided in 2025 (the most recently available case decision data) decreases precipitously over time. It is therefore likely that most undecided cases were dismissed. One potential reason for dismissal after assignment to an arbitrator is ineligibility as determined by the arbitrator. Arbitrators are responsible for assessing the eligibility of each case and dismissing cases ineligible for arbitration.

The main analytic sample consists of 677,137 independent dispute resolution cases initiated in 2023, of which 517,734 were decided before December 31, 2025. The 517,734 cases have a corresponding 893,725 unique line items (individual services). Case volumes and wait times are calculated using the counts of initiated cases; provider win rates, payments, and offers are calculated using the results from decided cases. The main outcomes of interest are case volumes for each method of selection, provider win rate, payment, and wait time in days. Appendix Figure A1 presents the share of cases with an arbitrator assigned using each selection method by quarter of filing. The share of cases in which an arbitrator is randomly assigned increases over time.

## B. Arbitrator Selection Process and Selection Methods

Arbitrators are chosen using the following process. The provider (the provider initiates more than 99% of cases[1], therefore the initiating party is referred to as provider and non-initiating party as insurer) proposes an arbitrator. The insurer can accept, reject, or not respond. If she accepts, then the arbitrator is jointly chosen; if she does not respond the provider's proposal is accepted by default; and if she rejects then she has her own chance to propose an arbitrator. If the insurer proposes an arbitrator, the provider gets a chance to take one of the three actions. If the provider accepts then the arbitrator is jointly chosen; if he does not respond the arbitrator is chosen by the insurer by default; and if he rejects, then an arbitrator is randomly assigned.

Both Agreed indicates both parties jointly chose the arbitrator; Default to Provider denotes an arbitrator proposed by the provider and a non-response from the insurer; Default to Insurer denotes an arbitrator proposed by the insurer and a non-response from the provider; Default to None indicates that neither party proposed any arbitrator; Random – COI indicates that the arbitrator initially selected attested to a conflict of interest with either provider or insurer, recused itself, and an arbitrator was randomly selected from the remaining pool; Random – Disagreed indicates that both parties rejected the other's proposal and an arbitrator was randomly assigned after both parties rejected each other's offers. Conflicts of interest are rare, occurring in fewer than 1% of cases. For cases randomly assigned due to a conflict of interest, only the provider-insurer pair that had a conflict of interest with an arbitrator and the re-assigned, unconflicted arbitrator is observed. Therefore, from this data, it is not straightforward to identify conflicted parties.

Providers initiate nearly all disputes, but this does not imply that their proposal must be accepted often. Table 2 shows that arbitrators vary in case outcomes. This could be due to unobserved factors such as case

composition or to true differences among arbitrators. It is reasonable to assume that most providers and insurers have good information about arbitrators, their provider win rates, payments, and wait times. It is also reasonable to assume that both disputing parties care about the expected payment (providers want a high payment, insurers want a low payment) and act in their best interest. If arbitrators truly differ in how they decide cases, then we would not expect insurers to accept an arbitrator that administers decisions adverse to their interests. It is thus surprising to observe insurers accepting high provider win rate, high payment arbitrators.

## C. Additional Information on Case Outcomes, Case Volumes, and Methods of Selection

The differences in case outcomes could result from dispute party offering behavior, case compositions, disputing party selection, or other factors. Appendix Table A4 presents mean payment, provider offer, insurer offer, payment in dollars, and fees for each of the 13 arbitrators deciding cases in 2023. Payment and offers are winsorized at the 99th percentile of line-items to mitigate the influence of outliers. Arbitrators charge the Single Fee for cases with one line item (single line item) and Batched Fee for cases with multiple line items. Mean insurer offers range from 124% (Keystone) of the QPA to 188% (Island Peer Review Organization, IPRO). Provider offers range from 435% (MPRO) to 1,065% (Keystone). The 64 percentage point range of mean insurer offers is small relative to the 630 percentage point range of mean provider offers and the 360 percentage point range of awards. Payment in dollars is available in only 497,426 out of 893,725 total line-items due to small cell size suppressions. Payment is winsorized at the line-item level, at the 99th percentile. Winsorized payments range from $675 for Medical Evaluators of Texas (MET) to $2,062 for Federal Hearings and Appeals Services (FHAS). Payment in dollars is reported at the metropolitan area by service level in the public use file. I use CMS' National Plan and Provider Enumeration System (NPPES) data (which includes NPI and zip code) [S3], the Department of Housing and Urban Development's Zip to Core Based Statistical Area (CBSA) crosswalk [S4], and the Environmental Protection Agency's CBSA file [S5] to match cases to metropolitan areas. Fees are presented for reference.

Case outcomes, especially payment, could be closely linked to arbitrator case compositions. Appendix Tables A5–A9 present the main table split by the following broad service categories: Radiology, Emergency, Anesthesia, Air Ambulance, and all other. Appendix Table A10 contains the definitions I use to classify disputes into broad service categories. Note that initiated but undecided cases do not have information about the type of health care service and therefore these tables omit them. Arbitrator mean case outcomes are different across services, but in general the gradient in win rates and payments persists.

Patterns in arbitrator case outcomes remain after splitting the data by selection method. Appendix Table A11 presents case outcomes and decision rates split by random (Random – Disagreed or Random – COI) and non-random assignment. The same gradient in case outcomes remains in both random and non-random subsamples.

Selection methods also vary across specific provider-insurer pairs. Appendix Table A12 presents the methods of selection used by the 19 provider-insurer pairs with the highest case volumes, together with all

remaining pairs combined.

## D. Arbitrator Tenure, Availability, and Capacity

Arbitrators vary widely in the number of total cases they decide, the number of randomly assigned cases they decide, and in the share of initiated cases they decide. It is important to know arbitrator capacity and availability during 2023. Are the observed differences in case volumes attributable to differences in arbitrator availability or differences in choices made by disputing parties? All 13 arbitrators were certified to accept cases for the duration of 2023. Unfortunately, I do not observe either capacity or availability well. From what is observable, all arbitrators seem to have been active for most months during 2023. I do not observe this with certainty and therefore capacity and availability remain a concern for my analysis.

Using the Internet Archive, I can observe some windows during which arbitrators were or were not unavailable. Each Internet Archive snapshot has a date on which it was taken and the date the page was last modified. From January 1 [S6] to January 28 [S7], two arbitrators were not accepting disputes and the other 11 were, according to the CMS website. Between February 6 [S8] and June 10 [S9], September 6 to September 7 [S10], and September 15 to October 4 [S11], all 13 were accepting. From January 29 to February 5, June 11 to September 5, September 8 to September 14, and October 5 to December 31, I do not know which were available and which were unavailable. The dispute resolution process was closed to new single line-item disputes between August 3 and October 6 and closed to new batched or air ambulance disputes between August 3 and December 15 [S12]. The Internet Archive only has snapshots covering 174 days in 2023. It could be that any of the 13 arbitrators were not accepting disputes for periods that would not be captured by the Internet Archive. A CMS Fact Sheet [S13] published in September 2025 states that severely backlogged arbitrators were temporarily prevented from accepting new disputes "over the past year." The timing of these actions is vague and whether CMS was clamping down on some arbitrator as early as 2023 is unknown. Keystone and PPR have the two lowest case volumes, but their unavailability windows cannot fully explain the discrepancy in case volumes among arbitrators.

I observe nothing about arbitrator capacities. Appendix Table A1 presents revenues, but this is likely too noisy to be very informative about each arbitrator's administrative capability. Ultimately, capacity and availability are important factors that I cannot observe. There is evidence that two arbitrators stopped accepting cases for roughly a month.

# Appendix Tables

## Appendix Table A1: Arbitrator Revenues and Corporate Structures

| Arbitrator | Revenue | Year | Parent Org | Structure |
|---|---|---|---|---|
| Keystone | Not found, >$250M (Acentra) | 2023 | Carlyle Group, via Acentra Health | PE |
| MET | <$5M | 2023 | None, independent | LLC |
| PRI | $21M | 2023 | Orono Group | PE |
| PPR | <$5M | 2023 | Nexus Enterprises | LLC |
| NMRI | <$5M | 2023 | None, independent | For-profit, private |
| NMRC | $18.7M, $750M (ExamWorks est.) | 2023, estimate | CVC Capital Partners, via ExamWorks | PE |
| MFS | $2.40B, $4.90B (Maximus Inc.) | 2023 | Maximus, Inc. | For-profit, public |
| EdiPhy | Not found, $24.2M (MedManagement) | 2023 | MedManagement LLC | LLC |
| MCMC | Not found, $750M (ExamWorks est.) | n/a, estimate | CVC Capital Partners, via ExamWorks | PE |
| FHAS | Not found | — | None, independent | LLC |
| C2C | $47.5M | 2023 | TMF Health Quality Institute | Non-profit |
| MPRO | $10.5M | 2023 | None, independent | Non-profit |
| IPRO | $83.1M | 2023 | None, independent | Non-profit |

**Notes:** Arbitrator abbreviations correspond to entity names as follows: Keystone: Keystone Peer Review Organization, Inc.; MET: Medical Evaluators of Texas; PRI: Provider Resources, Inc.; PPR: ProPeer Resources, LLC; NMRI: National Medical Reviews Inc.; NMRC: Network Medical Review Company; MFS: Maximus Federal Services, Inc.; EdiPhy: EdiPhy Advisors, LLC; MCMC: MCMC Services, LLC; FHAS: Federal Hearings and Appeals Services, Inc.; C2C: C2C Innovative Solutions Inc.; MPRO: iMPROve Health, formerly Michigan Peer Review Organization; IPRO: Island Peer Review Organization. Revenues and corporate structures are taken from several sources: Acentra revenue [S14]; Keystone parent company [S15]; MET revenue [S16]; PRI revenue [S17]; PRI parent company [S18]; PPR revenue [S19]; PPR parent company [S20]; NMRI revenue [S21]; NMRC revenue [S22]; NMRC parent company revenue [S23]; NMRC parent company [S24,S25]; MFS revenue and parent company [S26]; EdiPhy parent company revenue [S27]; EdiPhy parent company [S28]; MCMC parent company [S29],[14]; FHAS corporate structure [S30]; C2C revenue [S31]; C2C parent company [S32]; MPRO revenue [S33]; IPRO revenue [S34]. It was not possible to obtain full information for each arbitrator.

## Appendix Table A2: FOIA Arbitrator Identifiers Matched to CMS PUF Arbitrator Identifiers

| CertifiedIDREntity | Keystone | MET | PRI | PPR | NMRI | NMRC | MFS | EdiPhy | MCMC | FHAS | C2C | MPRO | IPRO |
|---|---|---|---|---|---|---|---|---|---|---|---|---|---|
| Keystone Peer Review Organization, Inc. | 6 | 0 | 0 | 0 | 0 | 0 | 0 | 0 | 0 | 0 | 0 | 0 | 0 |
| Medical Evaluators of Texas | 0 | 628 | 0 | 0 | 0 | 0 | 0 | 0 | 0 | 0 | 0 | 0 | 0 |
| Provider Resources, Inc. | 0 | 0 | 142 | 0 | 0 | 0 | 0 | 0 | 0 | 0 | 0 | 0 | 0 |
| ProPeer Resources, LLC | 0 | 0 | 0 | 31 | 0 | 0 | 0 | 0 | 0 | 0 | 0 | 0 | 0 |
| National Medical Reviews, Inc. | 0 | 0 | 0 | 0 | 220 | 0 | 0 | 0 | 0 | 0 | 0 | 0 | 0 |
| Network Medical Review Company, Ltd. | 0 | 0 | 0 | 0 | 0 | 16 | 0 | 0 | 0 | 0 | 0 | 0 | 0 |
| Maximus Federal Services, Inc. | 0 | 0 | 0 | 0 | 0 | 0 | 88 | 0 | 0 | 0 | 0 | 0 | 0 |
| EdiPhy Advisors, L.L.C. | 0 | 0 | 0 | 0 | 0 | 0 | 0 | 296 | 0 | 0 | 0 | 0 | 0 |
| MCMC Services, LLC | 0 | 0 | 0 | 0 | 0 | 0 | 0 | 0 | 429 | 0 | 0 | 0 | 0 |
| Federal Hearings and Appeals Services, Inc. | 0 | 0 | 1 | 0 | 0 | 0 | 0 | 0 | 0 | 54 | 0 | 0 | 0 |
| C2C Innovative Solutions, Inc. | 0 | 0 | 0 | 0 | 0 | 0 | 0 | 0 | 1 | 0 | 260 | 0 | 0 |
| iMPROve Health | 0 | 0 | 0 | 0 | 0 | 0 | 0 | 0 | 0 | 0 | 0 | 4 | 0 |
| Island Peer Review Organization | 0 | 0 | 0 | 0 | 0 | 0 | 0 | 0 | 0 | 0 | 0 | 0 | 13 |

**Notes:** Author's analysis of CMS independent dispute resolution reports and FOIA arbitrator identity information. Using the arbitrator identifiers given in the public use file for case decisions in the second half of 2025, I verify the FOIA arbitrator identifiers for cases initiated in 2023. For cases initiated in 2023 and decided in Q3 or Q4 of 2025, the identifiers match in 2,187 of 2,189 disputes.

**Appendix Table A3: Tabulation of Quarter and Year of Decision for Cases Initiated in 2023**

| Year | Q1 | Q2 | Q3 | Q4 |
|---|---|---|---|---|
| 2023 | 4,892 | 37,826 | 36,460 | 64,744 |
| 2024 | 119,347 | 79,885 | 77,878 | 69,720 |
| 2025 | 19,020 | 5,772 | 1,380 | 810 |

**Notes:** Author's analysis of IDR public use files and arbitrator assignment data obtained via FOIA. Cell counts are numbers of cases (initiated in 2023) decided in the given quarter.

## Appendix Table A4: Payments, Offers, and Fees by Arbitrator

| Arbitrator | Payment As % QPA | Provider Offer as % QPA | Insurer Offer as % QPA | Payment ($) | Single Fee ($) | Batched Fee ($) |
|---|---|---|---|---|---|---|
| Keystone | 610% | 1,065% | 124% | 1,988 | 600 | 800 |
| MET | 534% | 606% | 135% | 675 | 350 | 550 |
| PRI | 361% | 449% | 161% | 813 | 549 | 699 |
| PPR | 461% | 556% | 162% | 991 | 690 | 930 |
| NMRI | 340% | 455% | 144% | 1,089 | 490 | 770 |
| NMRC | 411% | 487% | 163% | 960 | 397 | 655 |
| MFS | 503% | 659% | 145% | 1,793 | 365 | 475 |
| EdiPhy | 508% | 535% | 185% | 981 | 700 | 938 |
| MCMC | 478% | 527% | 145% | 1,399 | 395 | 660 |
| FHAS | 635% | 717% | 128% | 2,062 | 395 | 490 |
| C2C | 700% | 811% | 157% | 1,805 | 648 | 938 |
| MPRO | 386% | 435% | 161% | 940 | 620 | 850 |
| IPRO | 528% | 573% | 188% | 1,102 | 700 | 930 |

**Notes:** Author's analysis of IDR public use files and arbitrator assignment data obtained via FOIA. Payments and offers are winsorized at the line-item level at the 99th percentile. Arbitrator abbreviations correspond to entity names as follows: Keystone: Keystone Peer Review Organization, Inc.; MET: Medical Evaluators of Texas; PRI: Provider Resources, Inc.; PPR: ProPeer Resources, LLC; NMRI: National Medical Reviews Inc.; NMRC: Network Medical Review Company; MFS: Maximus Federal Services, Inc.; EdiPhy: EdiPhy Advisors, LLC; MCMC: MCMC Services, LLC; FHAS: Federal Hearings and Appeals Services, Inc.; C2C: C2C Innovative Solutions Inc.; MPRO: iMPROve Health, formerly Michigan Peer Review Organization; IPRO: Island Peer Review Organization.

**Appendix Table A5: Case Outcomes and Volumes by Arbitrator for Radiology Services**

| Arbitrator | Provider Win Rate | Payment As % QPA | Wait Time | Total Cases | Random | Jointly Agreed | Default to Provider | Default to Insurer |
|---|---|---|---|---|---|---|---|---|
| Keystone | 54% | 455% | 250 | 440 | 0% | 18% | 3% | 79% |
| MET | 95% | 587% | 126 | 22,432 | 5% | 55% | 40% | 0% |
| PRI | 89% | 601% | 111 | 3,786 | 43% | 28% | 27% | 0% |
| PPR | 79% | 565% | 157 | 2,125 | 92% | 1% | 1% | 3% |
| NMRI | 60% | 455% | 148 | 2,917 | 67% | 21% | 6% | 4% |
| NMRC | 82% | 590% | 268 | 2,663 | 75% | 17% | 5% | 0% |
| MFS | 74% | 582% | 210 | 2,069 | 86% | 7% | 2% | 2% |
| EdiPhy | 97% | 653% | 173 | 10,443 | 5% | 39% | 56% | 0% |
| MCMC | 90% | 595% | 268 | 2,475 | 84% | 6% | 5% | 2% |
| FHAS | 91% | 787% | 42 | 8,015 | 13% | 29% | 58% | 0% |
| C2C | 74% | 916% | 113 | 3,645 | 30% | 40% | 4% | 25% |
| MPRO | 87% | 582% | 147 | 2,255 | 94% | 1% | 1% | 0% |
| IPRO | 96% | 679% | 234 | 5,125 | 21% | 17% | 61% | 0% |

**Notes:** Author's analysis of IDR public use files and arbitrator assignment data obtained via FOIA. Payments are winsorized at the line-item level at the 99th percentile. Arbitrator abbreviations correspond to entity names as follows: Keystone: Keystone Peer Review Organization, Inc.; MET: Medical Evaluators of Texas; PRI: Provider Resources, Inc.; PPR: ProPeer Resources, LLC; NMRI: National Medical Reviews Inc.; NMRC: Network Medical Review Company; MFS: Maximus Federal Services, Inc.; EdiPhy: EdiPhy Advisors, LLC; MCMC: MCMC Services, LLC; FHAS: Federal Hearings and Appeals Services, Inc.; C2C: C2C Innovative Solutions Inc.; MPRO: iMPROve Health, formerly Michigan Peer Review Organization; IPRO: Island Peer Review Organization. Cases may span more than one service type and can appear in more than one table. Service is unknown for initiated but undecided disputes, which do not appear in this table.

**Appendix Table A6: Case Outcomes and Volumes by Arbitrator for Emergency Services**

| Arbitrator | Provider Win Rate | Payment As % QPA | Wait Time | Total Cases | Random | Jointly Agreed | Default to Provider | Default to Insurer |
|---|---|---|---|---|---|---|---|---|
| Keystone | 51% | 275% | 232 | 2,224 | 0% | 48% | 9% | 43% |
| MET | 62% | 229% | 168 | 23,629 | 31% | 26% | 42% | 1% |
| PRI | 57% | 222% | 122 | 18,222 | 63% | 31% | 5% | 1% |
| PPR | 78% | 283% | 81 | 11,990 | 95% | 2% | 1% | 0% |
| NMRI | 76% | 263% | 153 | 25,738 | 34% | 38% | 25% | 3% |
| NMRC | 75% | 333% | 284 | 33,717 | 35% | 43% | 21% | 0% |
| MFS | 71% | 296% | 208 | 17,436 | 60% | 6% | 1% | 33% |
| EdiPhy | 93% | 311% | 195 | 18,347 | 63% | 23% | 12% | 1% |
| MCMC | 93% | 349% | 305 | 39,354 | 26% | 48% | 24% | 2% |
| FHAS | 90% | 353% | 44 | 44,024 | 14% | 42% | 44% | 0% |
| C2C | 85% | 299% | 99 | 33,294 | 24% | 29% | 29% | 17% |
| MPRO | 89% | 281% | 153 | 22,822 | 43% | 38% | 16% | 2% |
| IPRO | 86% | 363% | 217 | 23,687 | 20% | 40% | 40% | 0% |

**Notes:** Author's analysis of IDR public use files and arbitrator assignment data obtained via FOIA. Payments are winsorized at the line-item level at the 99th percentile. Arbitrator abbreviations correspond to entity names as follows: Keystone: Keystone Peer Review Organization, Inc.; MET: Medical Evaluators of Texas; PRI: Provider Resources, Inc.; PPR: ProPeer Resources, LLC; NMRI: National Medical Reviews Inc.; NMRC: Network Medical Review Company; MFS: Maximus Federal Services, Inc.; EdiPhy: EdiPhy Advisors, LLC; MCMC: MCMC Services, LLC; FHAS: Federal Hearings and Appeals Services, Inc.; C2C: C2C Innovative Solutions Inc.; MPRO: iMPROve Health, formerly Michigan Peer Review Organization; IPRO: Island Peer Review Organization. Cases may span more than one service type and can appear in more than one table. Service is unknown for initiated but undecided disputes, which do not appear in this table.

**Appendix Table A7: Case Outcomes and Volumes by Arbitrator for Anesthesia Services**

| Arbitrator | Provider Win Rate | Payment As % QPA | Wait Time | Total Cases | Random | Jointly Agreed | Default to Provider | Default to Insurer |
|---|---|---|---|---|---|---|---|---|
| Keystone | 56% | 332% | 256 | 511 | 0% | 29% | 1% | 70% |
| MET | 88% | 404% | 180 | 2,201 | 20% | 37% | 39% | 1% |
| PRI | 60% | 226% | 105 | 2,016 | 65% | 30% | 1% | 0% |
| PPR | 64% | 411% | 89 | 1,811 | 73% | 18% | 1% | 0% |
| NMRI | 84% | 257% | 158 | 2,833 | 42% | 48% | 4% | 3% |
| NMRC | 72% | 369% | 259 | 2,945 | 49% | 42% | 3% | 0% |
| MFS | 68% | 396% | 208 | 1,915 | 55% | 15% | 6% | 19% |
| EdiPhy | 97% | 366% | 179 | 2,077 | 36% | 50% | 8% | 0% |
| MCMC | 96% | 578% | 287 | 2,900 | 48% | 43% | 2% | 1% |
| FHAS | 89% | 596% | 50 | 4,531 | 17% | 49% | 32% | 0% |
| C2C | 90% | 555% | 83 | 4,372 | 31% | 37% | 26% | 5% |
| MPRO | 90% | 396% | 128 | 1,878 | 63% | 28% | 1% | 1% |
| IPRO | 93% | 466% | 197 | 1,431 | 32% | 41% | 23% | 1% |

**Notes:** Author's analysis of IDR public use files and arbitrator assignment data obtained via FOIA. Payments are winsorized at the line-item level at the 99th percentile. Arbitrator abbreviations correspond to entity names as follows: Keystone: Keystone Peer Review Organization, Inc.; MET: Medical Evaluators of Texas; PRI: Provider Resources, Inc.; PPR: ProPeer Resources, LLC; NMRI: National Medical Reviews Inc.; NMRC: Network Medical Review Company; MFS: Maximus Federal Services, Inc.; EdiPhy: EdiPhy Advisors, LLC; MCMC: MCMC Services, LLC; FHAS: Federal Hearings and Appeals Services, Inc.; C2C: C2C Innovative Solutions Inc.; MPRO: iMPROve Health, formerly Michigan Peer Review Organization; IPRO: Island Peer Review Organization. Cases may span more than one service type and can appear in more than one table. Service is unknown for initiated but undecided disputes, which do not appear in this table.

**Appendix Table A8: Case Outcomes and Volumes by Arbitrator for Air Ambulance Services**

| Arbitrator | Provider Win Rate | Payment As % QPA | Wait Time | Total Cases | Random | Jointly Agreed | Default to Provider | Default to Insurer |
|---|---|---|---|---|---|---|---|---|
| Keystone | 42% | 205% | 207 | 296 | 0% | 39% | 1% | 60% |
| MET | 80% | 412% | 271 | 484 | 61% | 8% | 6% | 13% |
| PRI | 67% | 301% | 184 | 537 | 64% | 21% | 5% | 3% |
| PPR | 36% | 250% | 149 | 443 | 77% | 9% | 2% | 6% |
| NMRI | 78% | 333% | 159 | 1,411 | 22% | 54% | 14% | 7% |
| NMRC | 72% | 384% | 284 | 853 | 41% | 21% | 11% | 23% |
| MFS | 70% | 364% | 202 | 779 | 36% | 25% | 3% | 31% |
| EdiPhy | 96% | 391% | 143 | 1,463 | 22% | 48% | 18% | 10% |
| MCMC | 95% | 435% | 306 | 1,539 | 21% | 16% | 18% | 43% |
| FHAS | 90% | 476% | 53 | 5,987 | 1% | 57% | 42% | 1% |
| C2C | 88% | 356% | 133 | 903 | 18% | 42% | 15% | 25% |
| MPRO | 92% | 351% | 139 | 563 | 58% | 15% | 2% | 18% |
| IPRO | 97% | 527% | 213 | 430 | 19% | 53% | 14% | 13% |

**Notes:** Author's analysis of IDR public use files and arbitrator assignment data obtained via FOIA. Payments are winsorized at the line-item level at the 99th percentile. Arbitrator abbreviations correspond to entity names as follows: Keystone: Keystone Peer Review Organization, Inc.; MET: Medical Evaluators of Texas; PRI: Provider Resources, Inc.; PPR: ProPeer Resources, LLC; NMRI: National Medical Reviews Inc.; NMRC: Network Medical Review Company; MFS: Maximus Federal Services, Inc.; EdiPhy: EdiPhy Advisors, LLC; MCMC: MCMC Services, LLC; FHAS: Federal Hearings and Appeals Services, Inc.; C2C: C2C Innovative Solutions Inc.; MPRO: iMPROve Health, formerly Michigan Peer Review Organization; IPRO: Island Peer Review Organization. Cases may span more than one service type and can appear in more than one table. Service is unknown for initiated but undecided disputes, which do not appear in this table.

**Appendix Table A9: Case Outcomes and Volumes by Arbitrator for Other Services**

| Arbitrator | Provider Win Rate | Payment As % QPA | Wait Time | Total Cases | Random | Jointly Agreed | Default to Provider | Default to Insurer |
|---|---|---|---|---|---|---|---|---|
| Keystone | 39% | 915% | 256 | 3,008 | 0% | 28% | 6% | 65% |
| MET | 69% | 1295% | 191 | 10,169 | 9% | 57% | 33% | 0% |
| PRI | 39% | 1021% | 117 | 3,382 | 36% | 45% | 16% | 1% |
| PPR | 61% | 1438% | 122 | 2,397 | 54% | 23% | 15% | 2% |
| NMRI | 42% | 824% | 160 | 4,476 | 25% | 50% | 17% | 6% |
| NMRC | 65% | 1195% | 288 | 3,695 | 35% | 50% | 11% | 1% |
| MFS | 66% | 1095% | 206 | 8,215 | 12% | 42% | 25% | 20% |
| EdiPhy | 90% | 1188% | 185 | 5,318 | 19% | 51% | 26% | 2% |
| MCMC | 84% | 1042% | 291 | 7,889 | 19% | 55% | 20% | 4% |
| FHAS | 81% | 1152% | 51 | 24,763 | 2% | 42% | 56% | 0% |
| C2C | 74% | 1668% | 115 | 12,962 | 6% | 54% | 18% | 21% |
| MPRO | 76% | 1134% | 136 | 2,305 | 61% | 22% | 9% | 3% |
| IPRO | 91% | 1328% | 268 | 2,982 | 22% | 39% | 36% | 1% |

**Notes:** Author's analysis of IDR public use files and arbitrator assignment data obtained via FOIA. Payments are winsorized at the line-item level at the 99th percentile. Arbitrator abbreviations correspond to entity names as follows: Keystone: Keystone Peer Review Organization, Inc.; MET: Medical Evaluators of Texas; PRI: Provider Resources, Inc.; PPR: ProPeer Resources, LLC; NMRI: National Medical Reviews Inc.; NMRC: Network Medical Review Company; MFS: Maximus Federal Services, Inc.; EdiPhy: EdiPhy Advisors, LLC; MCMC: MCMC Services, LLC; FHAS: Federal Hearings and Appeals Services, Inc.; C2C: C2C Innovative Solutions Inc.; MPRO: iMPROve Health, formerly Michigan Peer Review Organization; IPRO: Island Peer Review Organization. Cases may span more than one service type and can appear in more than one table. Service is unknown for initiated but undecided disputes, which do not appear in this table.

**Appendix Table A10: Broad Service Category Definitions**

| Broad service category | CPT/HCPCS codes or definition |
|---|---|
| Radiology | 70010–79999; G0279 |
| Emergency | 99281–99285; 99291–99292 |
| Anesthesia | 00100–00222; 00300–00352; 00400–00474; 00500–00580; 00600–00670; 00700–00797; 00800–00882; 00902–00952; 01112–01173; 01200–01274; 01320–01444; 01462–01522; 01610–01680; 01710–01782; 01810–01860; 01916–01942; 01958–01969; 01990–01999; 64400–64530 |
| Other | All other service codes in Emergency/Non-Emergency reports |
| Air ambulance | All disputes in Air Ambulance reports |

**Appendix Table A11: Case Outcomes by Random/Non-Random Arbitrator Selection**

| | Provider Win Rate | | Decision Rate | | Payment as % QPA | | Provider Offer as % QPA | | Insurer Offer as % QPA | |
|---|---|---|---|---|---|---|---|---|---|---|
| Arbitrator | Random | Non-Random | Random | Non-Random | Random | Non-Random | Random | Non-Random | Random | Non-Random |
| Keystone | 74% | 45% | 82% | 56% | 486% | 610% | 716% | 1066% | 113% | 124% |
| MET | 63% | 84% | 67% | 77% | 367% | 551% | 502% | 617% | 173% | 131% |
| PRI | 67% | 53% | 71% | 69% | 368% | 352% | 454% | 442% | 166% | 155% |
| PPR | 74% | 71% | 74% | 75% | 388% | 1212% | 468% | 1467% | 164% | 144% |
| NMRI | 75% | 67% | 69% | 67% | 364% | 325% | 476% | 441% | 172% | 127% |
| NMRC | 71% | 76% | 77% | 79% | 417% | 406% | 494% | 480% | 184% | 142% |
| MFS | 70% | 69% | 75% | 72% | 371% | 622% | 459% | 849% | 182% | 107% |
| EdiPhy | 88% | 96% | 85% | 88% | 416% | 557% | 450% | 578% | 167% | 194% |
| MCMC | 89% | 92% | 89% | 79% | 483% | 475% | 522% | 529% | 183% | 126% |
| FHAS | 86% | 87% | 90% | 85% | 367% | 670% | 401% | 758% | 143% | 126% |
| C2C | 88% | 79% | 77% | 70% | 391% | 783% | 450% | 911% | 130% | 164% |
| MPRO | 86% | 90% | 81% | 90% | 431% | 329% | 492% | 367% | 171% | 148% |
| IPRO | 92% | 88% | 70% | 68% | 506% | 535% | 535% | 584% | 239% | 172% |

**Notes:** Author's analysis of IDR public use files and arbitrator assignment data obtained via FOIA. Payments and offers are winsorized at the line-item level at the 99th percentile. Arbitrator abbreviations correspond to entity names as follows: Keystone: Keystone Peer Review Organization, Inc.; MET: Medical Evaluators of Texas; PRI: Provider Resources, Inc.; PPR: ProPeer Resources, LLC; NMRI: National Medical Reviews Inc.; NMRC: Network Medical Review Company; MFS: Maximus Federal Services, Inc.; EdiPhy: EdiPhy Advisors, LLC; MCMC: MCMC Services, LLC; FHAS: Federal Hearings and Appeals Services, Inc.; C2C: C2C Innovative Solutions Inc.; MPRO: iMPROve Health, formerly Michigan Peer Review Organization; IPRO: Island Peer Review Organization.

## Appendix Table A12: Selection Methods Used by Large Provider-Insurer Pairs in 2023

| Provider-Insurer Pair | Total Cases | Both Agreed | Default: Provider | Default: Insurer | Random COI | Random Disagreed |
|---|---|---|---|---|---|---|
| Team Health x UnitedHealthcare | 93,787 | 35% | 2% | 1% | 0% | 63% |
| Team Health x Anthem | 33,997 | 8% | 38% | 1% | 0% | 53% |
| Envision x UnitedHealthcare | 23,141 | 67% | 2% | 0% | 0% | 30% |
| Radiology Ptnrs x Aetna | 22,762 | 57% | 43% | 0% | 0% | 0% |
| Radiology Ptnrs x Multiplan | 20,027 | 6% | 46% | 3% | 0% | 45% |
| AGS Health x Anthem | 18,348 | 27% | 39% | 10% | 0% | 24% |
| SCP Health x Anthem | 17,402 | 29% | 29% | 16% | 0% | 26% |
| AGS Health x Multiplan | 13,539 | 8% | 28% | 3% | 0% | 61% |
| SCP Health x Multiplan | 12,885 | 6% | 31% | 19% | 0% | 44% |
| Team Health x BCBS TN | 11,810 | 1% | 99% | 0% | 0% | 1% |
| SCP Health x BCBS FL | 9,316 | 28% | 53% | 8% | 1% | 10% |
| AGS Health x BCBS FL | 9,218 | 46% | 46% | 3% | 1% | 4% |
| Team Health x Aetna | 8,243 | 81% | 19% | 0% | 0% | 0% |
| Sonoran Radiology x UnitedHealthcare | 6,378 | 17% | 35% | 1% | 0% | 47% |
| AGS Health x UnitedHealthcare | 5,992 | 67% | 2% | 0% | 0% | 30% |
| Radiology Ptnrs x UnitedHealthcare | 5,332 | 21% | 1% | 3% | 7% | 69% |
| Total Care x BCBS TX | 4,901 | 30% | 70% | 0% | 0% | 0% |
| Envision x Anthem | 4,873 | 50% | 18% | 1% | 0% | 30% |
| SCP Health x UnitedHealthcare | 4,786 | 59% | 3% | 9% | 0% | 28% |
| Other Pair | 350,400 | 41% | 26% | 9% | 2% | 22% |

**Notes:** Author's analysis of CMS independent dispute resolution public use files and CMS arbitrator identity and assignment data obtained using FOIA. The table includes the 19 provider-insurer pairs with the most disputes (initiated in 2023) decided, and all other pairs. Initiated but undecided cases do not have provider or insurer identity and are included under the Other Pair category. Selection method "Default to None" is omitted from this table because it occurs only in one case.

# Appendix Figures

**Appendix Figure A1: Selection Methods by Quarter of Filing**

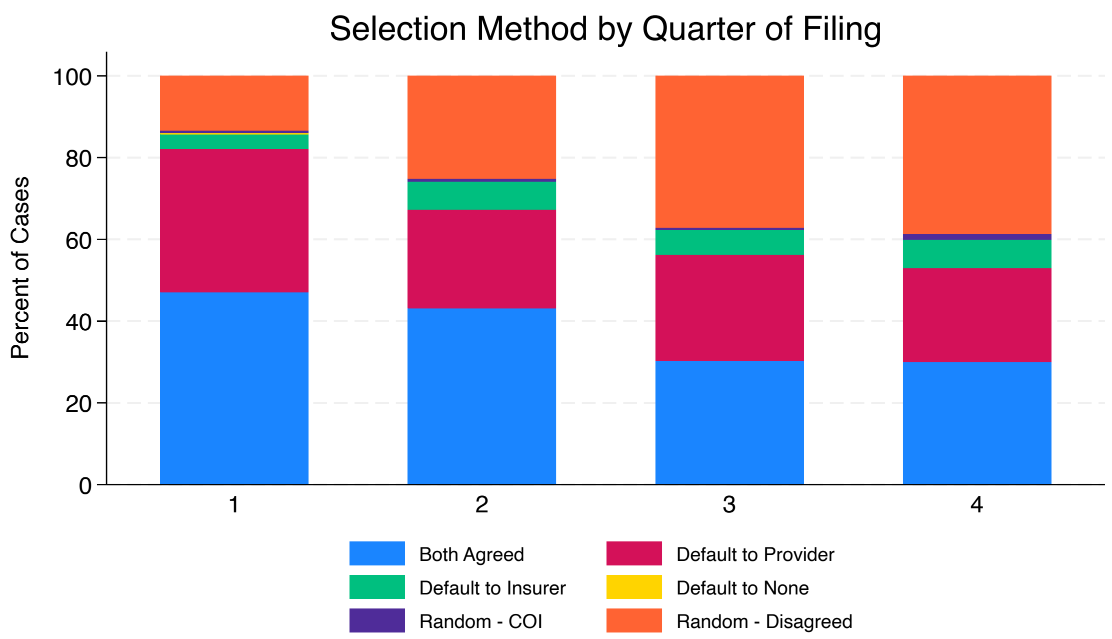


**Notes:** Author's analysis of Arbitrator Selection Method obtained via FOIA. I use quarterly counts of cases from [S1] to determine quarter of initiation for each case. For example, 136,111 cases were initiated in quarter 1 of 2023 and the first dispute in the FOIA data is number 200,502. I then assign cases with dispute numbers between 200,502 and 336,612 to quarter 1.

# Appendix References


[S1] Rosso RJ, Shen WW. *No Surprises Act (NSA) Independent Dispute Resolution (IDR) Process Data Analysis for 2023.* Washington (DC): Congressional Research Service; 2025 Jan 17 [accessed 2026 Jul 29]. Report No. R48359. Available from: `https://www.congress.gov/crs-product/R48359`

[S2] Centers for Medicare & Medicaid Services. List of certified independent dispute resolution entities [Internet]. Archived at the Internet Archive [accessed 2026 Jul 29]. Available from: `https://web.archive.org/web/20230112221757/https://www.cms.gov/nosurprises/help-resolve-payment-disputes/certified-idre-list`

[S3] Centers for Medicare & Medicaid Services. National Plan and Provider Enumeration System (NPPES) downloadable file, September 2025 [Internet]. Baltimore (MD): CMS; 2025 Sep [accessed 2025 Sep 12]. Available from: `https://www.cms.gov/medicare/regulations-guidance/administrative-simplification/data-dissemination`

[S4] US Department of Housing and Urban Development. HUD USPS ZIP code crosswalk files: ZIP–CBSA, 2023 quarter 2 [Internet]. Washington (DC): HUD; 2023 [accessed 2025 Sep 18]. Available from: `https://www.huduser.gov/apps/public/uspscrosswalk/home`

[S5] US Environmental Protection Agency. Air Quality System code tables: core based statistical areas (CBSAs) [Internet]. Washington (DC): EPA; [accessed 2025 Sep 18]. Available from: `https://aqs.epa.gov/aqsweb/documents/codetables/cbsas.html`

[S6] Centers for Medicare & Medicaid Services. List of certified independent dispute resolution entities [Internet]. Baltimore (MD): CMS; 2022 Dec 30 [accessed 2026 Sep 19]. Available from: `https://web.archive.org/web/20221230151105/https://www.cms.gov/nosurprises/help-resolve-payment-disputes/certified-idre-list`

[S7] Centers for Medicare & Medicaid Services. List of certified independent dispute resolution entities [Internet]. Baltimore (MD): CMS; 2023 Jan 28 [accessed 2026 Sep 19]. Available from: `https://web.archive.org/web/20230128165627/https://www.cms.gov/nosurprises/help-resolve-payment-disputes/certified-idre-list`

[S8] Centers for Medicare & Medicaid Services. List of certified independent dispute resolution entities [Internet]. Baltimore (MD): CMS; 2023 Feb 16 [accessed 2026 Sep 19]. Available from: `https://web.archive.org/web/20230216150958/https://www.cms.gov/nosurprises/help-resolve-payment-disputes/certified-idre-list`

[S9] Centers for Medicare & Medicaid Services. List of certified independent dispute resolution entities [Internet]. Baltimore (MD): CMS; 2023 Jun 10 [accessed 2026 Sep 19]. Available from: `https://web.archive.org/web/20230610142621/https://www.cms.gov/nosurprises/help-resolve-payment-disputes/certified-idre-list`

[S10] Centers for Medicare & Medicaid Services. List of certified organizations [Internet]. Baltimore (MD): CMS; 2023 Sep 7 [accessed 2026 Sep 19]. Available from: https://web.archive.org/web/20230907020946/https://www.cms.gov/priorities/legislation/no-surprises-act/list-certified-organizations

[S11] Centers for Medicare & Medicaid Services. List of certified independent dispute resolution entities [Internet]. Baltimore (MD): CMS; 2023 Oct 4 [accessed 2026 Sep 19]. Available from: https://web.archive.org/web/20231004155546/https://www.cms.gov/nosurprises/help-resolve-payment-disputes/certified-idre-list

[S12] Centers for Medicare & Medicaid Services. Notices [Internet]. Baltimore (MD): CMS; [accessed 2026 Sep 19]. Available from: https://www.cms.gov/initiatives/no-surprise-billing/overview/notices

[S13] Centers for Medicare & Medicaid Services. Fact sheet: clearing the independent dispute resolution backlog [Internet]. Baltimore (MD): CMS; 2025 Sep 19 [accessed 2026 Sep 19]. Available from: https://www.cms.gov/files/document/fact-sheet-clearing-independent-dispute-resolution-backlog.pdf

[S14] Acentra Health. Acentra Health named Corporate Growth Company of the Year [Internet]. 2024 [accessed 2026 Sep 18]. Available from: https://acentra.com/news/acentra-health-corporate-growth-company-of-the-year-by-acg-national-capital

[S15] Carlyle Group. Acentra Health [Internet]. [accessed 2026 Sep 18]. Available from: https://www.carlyle.com/our-business/portfolio-of-investments/acentra-health

[S16] ZoomInfo. MET Healthcare Solutions [Internet]. 2023 [accessed 2026 Sep 18]. Available from: https://www.zoominfo.com/c/met-healthcare-solutions/455252013

[S17] ZoomInfo. Provider Resources [Internet]. 2023 [accessed 2026 Sep 18]. Available from: https://www.zoominfo.com/c/provider-resources-inc/124142118

[S18] LinkedIn. Orono Group [Internet]. [accessed 2026 Sep 18]. Available from: https://www.linkedin.com/company/oronogroup/

[S19] ZoomInfo. ProPeer Resources [Internet]. 2023 [accessed 2026 Sep 18]. Available from: https://www.zoominfo.com/c/propeer-resources-inc/31473666

[S20] ProPeer Resources. Who we are [Internet]. [accessed 2026 Sep 18]. Available from: https://propeer.com/who-we-are/

[S21] ZoomInfo. National Medical Reviews [Internet]. 2023 [accessed 2026 Sep 18]. Available from: https://www.zoominfo.com/c/national-medical-reviews-inc/190235037

[S22] ZoomInfo. NMR [Internet]. 2023 [accessed 2026 Sep 18]. Available from: https://www.zoominfo.com/c/nmr-co/40016264

[S23] RocketReach. ExamWorks profile [Internet]. [accessed 2026 Sep 18]. Available from: https://rocketreach.co/examworks-profile_b59b43b3f69d4e5b

[S24] PitchBook. NMR: company profile [Internet]. [accessed 2026 Sep 18]. Available from: https://pitchbook.com/profiles/company/51172-48#faqs

[S25] MedRecords.ai. ExamWorks review [Internet]. 2026 [accessed 2026 Sep 18]. Available from: https://medrecords.ai/reports/examworks-review/

[S26] Maximus Inc. Maximus reports fourth quarter and full year results for fiscal year 2023 [Internet]. 2023 [accessed 2026 Sep 18]. Available from: https://investor.maximus.com/news-events/press-releases/detail/529/maximus-reports-fourth-quarter-and-full-year-results-for

[S27] Growjo. MedManagement: revenue, competitors, alternatives [Internet]. [accessed 2026 Sep 18]. Available from: https://growjo.com/company/MedManagement

[S28] LinkedIn. MedManagement LLC [Internet]. [accessed 2026 Sep 18]. Available from: https://www.linkedin.com/company/medmanagement-llc/about/

[S29] Sweetspot. MCMC Services LLC [Internet]. [accessed 2026 Sep 18]. Available from: https://www.sweetspot.so/markets/federal/contractors/mcmc-services-llc-xfz2t4qzeku8/

[S30] Federal Hearings and Appeals Services. FHAS earns NCQA accreditation for utilization management [Internet]. [accessed 2026 Sep 18]. Available from: https://www.fhas.com/resources/industry-updates/for-immediate-release-fhas-earns-ncqa-accreditation-for-utilization-management/

[S31] ProPublica. C2C Innovative Solutions Inc [Internet]. 2023 [accessed 2026 Sep 18]. Available from: https://projects.propublica.org/nonprofits/organizations/473641754

[S32] TMF Health Quality Institute. Program integrity [Internet]. [accessed 2026 Sep 18]. Available from: https://tmf.org/Our-Work/Program-Integrity

[S33] ProPublica. Improve Health (MPRO) [Internet]. 2023 [accessed 2026 Sep 18]. Available from: https://projects.propublica.org/nonprofits/organizations/382536610

[S34] ProPublica. Island Peer Review Organization Inc [Internet]. 2023 [accessed 2026 Sep 18]. Available from: https://projects.propublica.org/nonprofits/organizations/112662689
